# The Model Organism *Hermissenda crassicornis* (Gastropoda: Heterobranchia) Is a Species Complex

**DOI:** 10.1371/journal.pone.0154265

**Published:** 2016-04-22

**Authors:** Tabitha Lindsay, Ángel Valdés

**Affiliations:** Department of Biological Sciences, California State Polytechnic University, Pomona, California, United States of America; Tokai University, JAPAN

## Abstract

*Hermissenda crassicornis* is a model organism used in various fields of research including neurology, ecology, pharmacology, and toxicology. In order to investigate the systematics of this species and the presence of cryptic species in *H*. *crassicornis*, we conducted a comprehensive molecular and morphological analysis of this species covering its entire range across the North Pacific Ocean. We determined that *H*. *crassicornis* constitutes a species complex of three distinct species. The name *Hermissensa crassicornis* is retained for the northeast Pacific species, occurring from Alaska to Northern California. The name *H*. *opalescens* is reinstated for a species occurring from the Sea of Cortez to Northern California. Finally, the name *H*. *emurai* is maintained for the northwestern species, found in Japan and in the Russian Far East. These three species have consistent morphological and color pattern differences that can be used for identification in the field.

## Introduction

The repeatability of experiments involving living organisms heavily relies on the accuracy of species identifications. For instance, if separate studies on the same model organism use specimens that actually belong to different taxa, the results of those studies may not be comparable. Taxonomic accuracy is generally not an issue when dealing with laboratory strains or model species raised in captivity for generations such as *Caenorhabditis elegans*, *Drosophila melanogaster*, or *Aplysia californica*, but it can be important when research animals are collected in the field.

*Hermissenda crassicornis* (Eschscholtz, 1831) is an important model organism in neuroscience, including studies on classical conditioning [1–3], memory consolidation and associative learning [4–8], the structure of neural circuits [9–10] and neural physiology [11–13]. Additionally, *H*. *crassicornis* has been used to investigate ultrastructure and anatomy [14–15], larval and reproductive ecology [16–17], behavioral ecology [18–20] and pharmacology and toxicology [21–22], resulting in a wealth of papers and information widely cited in modern literature. Because *H*. *crassicornis* has an unusually broad geographic range, across the North Pacific Ocean [23], specimens collected for applied studies have diverse origins, typically from different locations between Southern California and Washington, but also from Russia. In many cases specimens were purchased from commercial suppliers and their exact origin is unknown or difficult to determine.

The taxonomy of *H*. *crassicornis* has not been reviewed for decades. In 1922 O’Donoghue [24] concluded that *Hermissenda opalescens* (Cooper, 1863), originally described from San Diego, California was a junior synonym of *H*. *crassicornis*, originally described from Sitka, Alaska, and this opinion became universally accepted [23, 25]. More recently the Japanese species *Cuthona emurai* Baba, 1937 was synonymized with *H*. *crassicornis* [25], establishing the currently recognized transpacific range for this species.

Recent integrative taxonomic studies have revealed that other widely distributed species of nudibranchs resulted to be species complexes composed of multiple species with much more restricted ranges [26–28]. In this paper we use similar methodologies to examine the genetic structure and morphological variation of *H*. *crassicornis* over its entire range in an attempt to determine the validity of previously described species. For this purpose we use a combination of molecular phylogenetics (based on four genes), species delimitation analyses, population genetics, and morphological comparisons.

## Materials and Methods

### Source of Specimens

All *Hermissenda crassicornis* specimens were obtained through SCUBA, on floating docks or during low tide by the authors or donated by colleagues. Specimens from California were collected under California Department of Fish and Game permit SC-9153. Specimens from Japan were collected under the permits of the Mouran and Oshoro Marine Stations. Specimens obtained by the authors were photographed and preserved in 95% ethanol. Specimens were deposited in the Cal Poly Pomona Invertebrate Collection (CPIC) and the Natural History Museum of Los Angeles County (LACM). Sequences of *Dondice occidentalis*, *Nayuca sebastiani*, *Godiva quadricolor*, and *Phyllodesmium jakobsenae* were obtained from Genbank and included in the analysis for comparison. Specimens of *Phidiana lascrusensis* were obtained from the Natural History Museum of Los Angeles County (LACM) and sequenced to be used as the outgroup.

### Morphological Analyses

At least three specimens of each clade were dissected using a Leica EZ4D stereo microscope. The buccal mass was extracted through a ventral incision and placed into a 10% NaOH solution for approximately 1 hour. The jaws were then removed from the buccal mass and placed in DI water for 5–10 minutes to remove excess NaOH. The jaws were then mounted, with masticatory boarder showing on an SEM stub. The remaining buccal mass was left in the 10% sodium hydroxide solution for 2–3 days to fully dissolve the tissue. The radula was then carefully removed from the solution and placed into DI water for 5–10 minutes to remove excess NaOH. The radula was then mounted on an SEM stub. SEM images were taken with a Hitachi S-3000N variable pressure scanning electron microscope.

### DNA Extraction, Amplification and Sequencing

A total of 42 specimens were sequenced for this study (Table 1), collected from several localities across the range of *Hermissenda crassicornis*. A combination of four gene fragments were sequenced for this project: mitochondrial 16S and COI and nuclear H3 and 18S.

**Table 1:** Specimens sequenced for this study, including locality, collector, museum voucher number, isolate code, and GenBank accession numbers (H3, 16S, COI, 18S).

Species Name	Location	Date collected	Voucher number	Isolate	H3	16S	CO1	18S
*Hermissenda crassicornis*	Alaska	-	-	-	-	-	KF643647	-
*Hermissenda crassicornis*	Alaska	-	-	-	-	-	KF6438398	-
*Hermissenda crassicornis*	Alaska	-	-	-	-	-	KF644184	-
*Hermissenda crassicornis*	Canada	-	-	-	-	-	KF643853	-
*Hermissenda crassicornis*	Canada	-	-	-	-	-	KF644243	-
*Hermissenda crassicornis*	Gig Harbor, WA	7/23/2014	CPIC-01102	TL191	KU950198	KU950107	KU950160	KU950132
*Hermissenda crassicornis*	Gig Harbor, WA	7/23/2014	CPIC-01103	TL192	KU950199	-	KU950161	KU950133
*Hermissenda crassicornis*	Lane County, OR	8/6/1971	LACM 71–87	TL160	KU950200	KU950108	KU950162	KU950134
*Hermissenda crassicornis*	Lane County, OR	8/6/1971	LACM 71–87	TL161	KU950201	KU950109	KU950163	KU950135
*Hermissenda crassicornis*	Point Reyes, CA	08/12/10	CPIC-00457	TL271	KU950202	KU950110	KU950164	KU950136
*Hermissenda crassicornis*	Sitka, AK	3/25/2014	CPIC-00959	TL062	KU950203	KU950111	KU950165	KU950137
*Hermissenda crassicornis*	Sitka, AK	3/25/2014	CPIC-00960	TL063	KU950204	KU950112	KU950166	KU950138
*Hermissenda crassicornis*	Victoria, BC, Canada	7/19/2014	CPIC-01104	TL193	KU950205	KU950113	KU950167	KU950139
*Hermissenda crassicornis*	Victoria, BC, Canada	7/19/2014	CPIC-01105	TL194	KU950206	KU950114	KU950168	KU950140
*Hermissenda crassicornis*	Victoria, BC, Canada	7/19/2014	CPIC-01106	TL195	KU950207	KU950115	KU950169	KU950141
*Hermissenda crassicornis*	Victoria, BC, Canada	7/19/2014	CPIC-01107	TL196	KU950208	-	KU950170	KU950142
*Hermissenda crassicornis*	Victoria, BC, Canada	7/19/2014	CPIC-01108	TL197	-	-	KU950171	KU950143
*Hermissenda crassicornis*	Victoria, BC, Canada	7/19/2014	CPIC-01109	TL198	-	KU950116	KU950172	KU950144
*Hermissenda crassicornis*	Victoria, BC, Canada	7/19/2014	CPIC-01110	TL199	KU950209	KU950117	KU950173	KU950145
*Hermissenda crassicornis*	Victoria, BC, Canada	7/19/2014	CPIC-01111	TL200	KU950210	KU950118	KU950174	KU950146
*Hermissenda crassicornis*	Victoria, BC, Canada	7/19/2014	CPIC-01112	TL201	KU950211	KU950119	KU950175	KU950147
*Hermissenda crassicornis*	Victoria, BC, Canada	7/20/2014	CPIC-01113	TL202	-	KU950120	KU950176	KU950148
*Hermissenda crassicornis*	Victoria, BC, Canada	7/20/2014	CPIC-01114	TL203	-	-	KU950177	-
*Hermissenda crassicornis*	Victoria, BC, Canada	7/20/2014	CPIC-01115	TL204	KU950212	KU950121	KU950178	KU950149
*Hermissenda crassicornis*	Victoria, BC, Canada	7/20/2014	CPIC-01116	TL205	-	-	KU950179	-
*Hermissenda emurai*	Muroran-Hokkaido, Japan	09/10/14	CPIC-01257	TL259	-	-	KU950180	-
*Hermissenda emurai*	Muroran-Hokkaido, Japan	09/10/14	CPIC-01258	TL260	-	-	KU950181	-
*Hermissenda emurai*	Muroran-Hokkaido, Japan	09/10/14	CPIC-01259	TL261	-	-	KU950182	-
*Hermissenda emurai*	Muroran-Hokkaido, Japan	09/10/14	CPIC-01260	TL262	KU950213	-	KU950183	KU950150
*Hermissenda emurai*	Muroran-Hokkaido, Japan	09/10/14	CPIC-01261	TL263	-	-	KU950184	-
*Hermissenda emurai*	Tateyama, Chiba, Japan	3/1/2014	CPIC-01080	TL184	KU950214	KU950122	KU950185	-
*Hermissenda emurai*	Tateyama, Chiba, Japan	3/1/2014	CPIC-01081	TL185	KU950215	KU950123	KU950186	KU950151
*Hermissenda opalescens*	Bahía de los Ángeles, Mexico	5/10-11/1976	LACM 76–1	TL154	KU950216	-	KU950187	-
*Hermissenda opalescens*	Bahía de los Ángeles, Mexico	5/10-11/1976	LACM 76–1	TL155	KU950217	-	KU950188	-
*Hermissenda opalescens*	Bahía de los Ángeles, Mexico	12/16/14	CPIC-01271	TL276	KU950218	KU950124	KU950189	KU950152
*Hermissenda opalescens*	Bodega Bay, CA	09/11/09	CPIC-00565	TL272	KU950219	KU950125	KU950190	KU950153
*Hermissenda opalescens*	Bodega Bay, CA	09/11/09	CPIC-00565	TL273	KU950220	KU950126	KU950191	KU950154
*Hermissenda opalescens*	Long Beach Marina, CA	6/21/2007	LACM 2007–2.2	TL143	KU950221	KU950127	KU950192	KU950155
*Hermissenda opalescens*	Long Beach Marina, CA	05/22/10	CPIC-00421	TL269	KU950222	KU950128	KU950193	-
*Hermissenda opalescens*	Long Beach Marina, CA	11/11/08	CPIC-00566	TL274	KU950223	-	KU950194	KU950156
*Hermissenda opalescens*	Malibu, CA	07/02/14	CPIC-01270	TL275	KU950224	KU950129	KU950195	KU950157
*Hermissenda opalescens*	Monterey Bay, CA	03/22/11	CPIC-00424	TL270	KU950225	KU950130	KU950196	KU950158
*Phidiana lascrucensis*	San Carlos, Mexico	May-97	LACM 25017	TL291	KU950226	KU950131	KU950197	KU950159
*Phyllodesmium jakobsenae*	-	-	-	-	HQ010456	HQ010524	HQ010489	GU339162
*Dondice occidentalis*	Bocas del Toro, Panama	-	LACM 2003–41.5	-	JQ699394	JQ699482	JQ699570	-
*Nayuca sebastiani*	St. Thomas, Virgin Is.	-	CPIC-00522		JQ699469	JQ699557	JQ699633	-
*Godiva quadricolor*	Knysna, South Africa	-	CASIZ 176385		HM162589	HM162680	HM162756	-

Legend: (-) no data available. Abbreviations: AK, Alaska; BC, British Columbia; CA, California; OR, Oregon; WA, Washington.

DNA extraction was performed using DNeasy Blood and Tissue Kit (Qiagen) or standard Chelex extraction. A small 1mm piece of tissue was cut from the foot or mantle or used from tissue samples and macerated using a sterilized razor blade. For Chelex extraction, the macerated tissue was transferred using sterilized forceps into a 1.7mL microcentrifuge tube containing 1mL of 1X TE Buffer and placed on a rotation block for at least 20 minutes to rehydrate the preserved tissue and allow cells to begin disassociating. Samples were then removed from the rotation block, vortexed for roughly 5 seconds, and centrifuged at 23,897.25 g for 3 minutes. Next, 975μL of 1X TE Buffer was removed from each sample being careful to not disturb the pellet of tissue in each tube. 175μL of 10% Chelex solution was added to each sample and vortexed. The samples were then placed in a 56°C hot water bath for 20 minutes. Samples were removed, vortexed for roughly 5 seconds and placed into a 100°C heat block for exactly 8 minutes. Each sample was vortexed for roughly 5 seconds and then centrifuged at 23,897.25 g for 3 minutes. The resultant supernatant was used for PCR. For Dneasy extraction, the manufacturer’s protocol for tissue samples was followed. The end products were used for PCR amplification.

The polymerase chain reaction (PCR) was used to amplify portions of the mitochondrial cytochrome c oxidase 1 (COI) and 16S ribosomal RNA (16S) genes, as well as the nuclear histone 3 (H3) gene and the first 500bp of 18S ribosomal RNA (18S) gene. The following universal primers were used to amplify the regions of interest for all specimens: COI (LCOI490 5’-GGTCAACAAATCATAAAGATATTGG-3’, HCO2198 5’TAAACTTCAGGGTGACCAAAAAATCA-3’) [29], 16S rRNA (16S ar-L 5’-CGCCTGTTTATCAAAAACAT-3’, 16S br-H 5’-CCGGTCTGAACTCAGATCACGT-3’) [30], H3 (H3 AF 5’-ATGGCTCGTACCAAGCAGACGGC-3’, H3 AR 5’-ATATCCTTGGGCATGATGGTGAC-3’) [31], and 18S (18SA1 5’-CTGGTTGATCCTGCCACTCATATGC-3’, 18S700R 5’-CGCGGCTGCTGGCACCAGAC -3’) [32]. Amplification of DNA was confirmed using agarose gel electrophoresis with ethidium bromide to detect the presence of DNA. PCR products were sent to Source Bioscience Inc. (Santa Fe Springs, CA, USA) for sequencing.

### Phylogenetic Analyses

Sequences were assembled, edited, and aligned using Geneious Pro 8.1 [33]. The Akaike information criterion [34] was executed in jModelTest [35] to determine the best-fit model of evolution for each gene (COI and 16S were portioned by codon position): GTR + I (H3 and COI 1^st^-2^nd^ codon positions), GTR + G (H3 3^rd^ codon positions), HKY + G (COI 3^rd^ codon positions), GTR+I+G (18S, 16S), and GTR+I+G for the entire concatenated dataset. Phylogenetic analyses were conducted with *Phidiana lascrucensis* as the outgroup and using a limited number of specimens of *H*. *crassicornis* for which all four genes were available. Maximum likelihood analyses were conducted for the entire concatenated alignment with RaXML [36] with 10,000 bootstrap repetitions and the GAMMAGI model (no partitions). Bayesian analyses were run in BEAST 1.8.2 [37], partitioned by gene and codon position (unlinked), with two runs of six chains for 10 million iterations with a sampling interval of 1,000 iterations and burn-in of 10%.

### Automatic Barcode Gap Discovery (ABGD) Analysis

ABGD analysis was run on the ingroup sequences to provide further corroboration for the delimitation of species identified through the phylogenetic and morphological analyses. ABGD infers the number of species present in a set of sequence data (and assigns individuals to the putative species) based on gaps in the distribution of pairwise distances between each sequence in a dataset [38]. The analysis was run twice for each gene individually, once using Kimura 2-parameter (K2) and once using Tamura-Nei (TN) distance matrices. The matrices were loaded into the online ABGD webtool (http://wwwabi.snv.jussieu.fr/public/abgd/abgdweb.html). The default relative gap width (x) of 1.5 and a range of prior values for maximum divergence of intraspecific diversity (*P*) from 0.001 to 0.1 were used.

### Haplotype Network and Population Genetics Analyses

A haplotype network was constructed for CO1 using TCS 1.21 [39]. Genetic structure of populations was analyzed in Arlequin [40] using analysis of molecular variance (AMOVA) and to test for genetic differentiation between populations (F_ST_). Two groups (eastern Pacific and western Pacific) were run using 7 populations (see Table 2) and three groups (Sea of Japan, northeastern Pacific and southeastern Pacific) using the same 7 populations. Significance of the AMOVA and Φ_ST_ analyses was tested using 16,000 permutations. AMOVA is a hierarchical approach analogous to ANOVA where the correlations among haplotypes at various hierarchical levels are used as F-statistics analogs. AMOVA computes the proportion of variation among groups (F_CT_), the proportion of variation among populations within groups (F_SC_) and the proportion of variation within populations (F_ST_).

**Table 2:** Simplified sequence groupings for AMOVA and F_ST_ Analysis determined based on geographic distribution.

Population	Number of specimens	Isolate
Washington	3	TL191, TL192, GQ292054
South East	6	TL143, TL154, TL274-TL276, TL269
South Japan	2	TL184, TL185
North Japan	5	TL259-TL263
North East	5	TL161, TL270-TL273
Canada	15	KF643853, KF644243, TL193-TL205
Alaska	5	KF643647, KF643898, KF644184, TL062, TL063

## Results

### Phylogenetic Analyses

**Fig 1:**
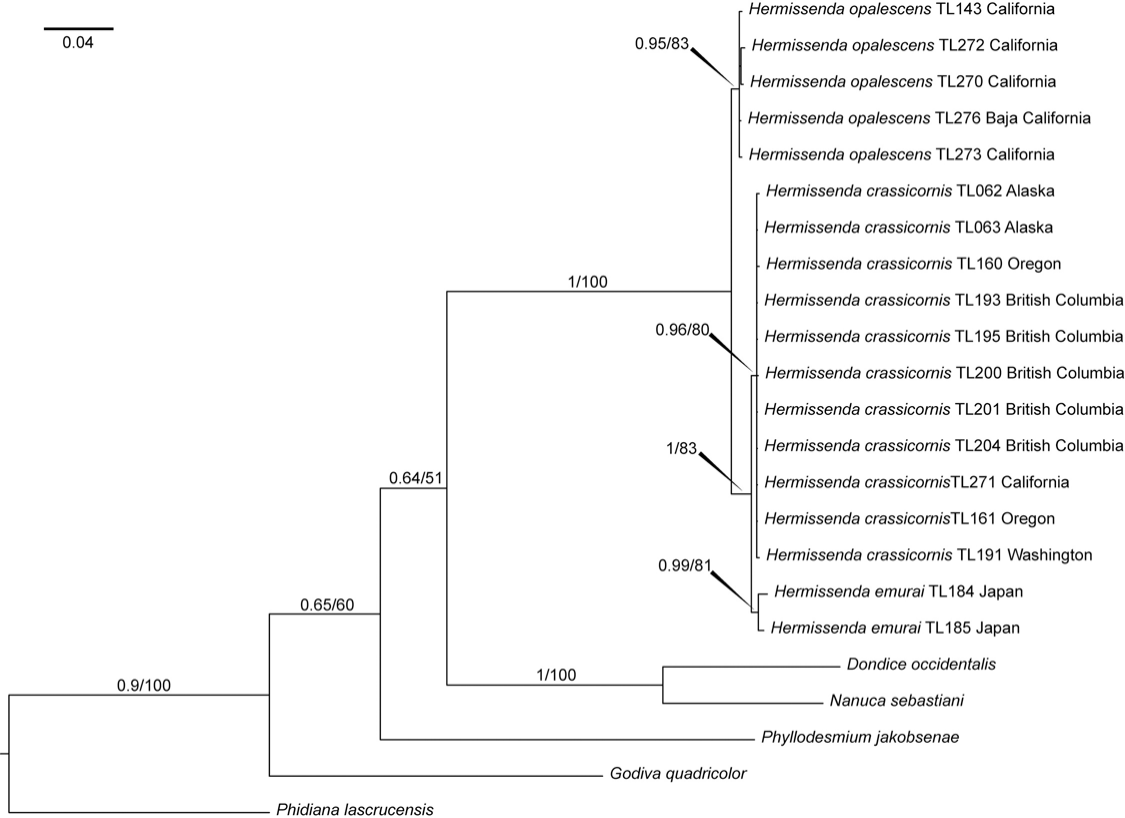
Bayesian consensus tree of the concatenated analysis including posterior probabilities (pp) and bootstrap values from the maximum likelihood (mlb) analysis. Only values >0.5 (pp) or 50 (mlb) are provided.

Bayesian and maximum likelihood consensus trees (Fig 1) have similar topologies and recovered the same clades. Bayesian pp values greater than or equal to 0.95 and mlb values greater than or equal to 70 were considered significant [41–42]. Specimens previously identified as *Hermissenda crassicornis* are split into three main clades in both trees. One clade includes specimens with a restricted range from the Sea of Japan [pp = 0.99; mlb = 81]. A second clade covers specimens with a distribution from Alaska through northern California [pp = 0.96; mlb = 80]. The third clade includes specimens with a range from northern California through the Sea of Cortez [pp = 0.95; mlb = 83].

### Automatic Barcode Gap Discovery (ABGD) Analysis

Using both K2 and TN distance matrices, the CO1 sequence showed a major barcode gap between a priori genetic distance thresholds of 0.01 and 0.02. Using a value of *P* between this range (.0129), three species were identified for CO1. Assignment of individuals within the three groups for CO1 matched the Bayesian and maximum likelihood phylogenies.

### Haplotype Network and Population Genetics Analyses

**Fig 2:**
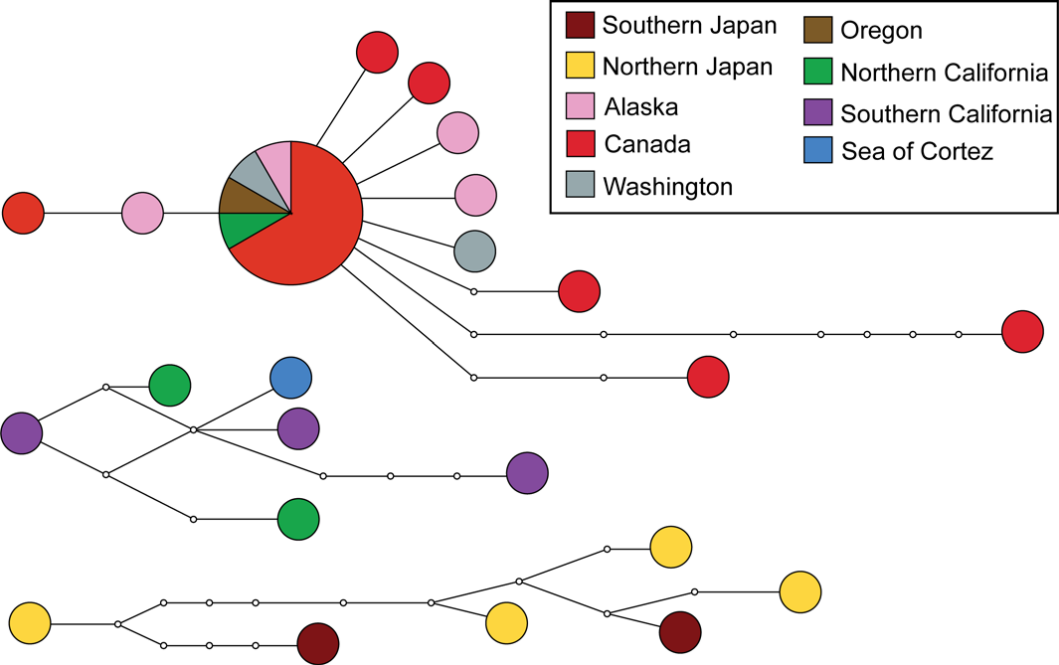
Haplotype network showing three distinct groups. Each circle represents a unique haplotype and the size of each circle indicates how many specimens share that haplotype, the larger the circle the more specimens sharing an identical haplotype. Each line between haplotypes indicates a single nucleotide polymorphism.

The haplotype network was unable to resolve all samples of CO1 of *Hermissenda crassicornis* specimens into a single network, suggesting the presence of more than one species. The analysis resolved three distinct haplotype networks (Fig 2). The sample composition of the three networks coincides with the three clades recovered in the phylogenetic analysis and the three species found in the species delimitation analysis. An AMOVA analysis was run to compare the genetic structure of western Pacific and eastern Pacific populations, and again the groups resolved in the Bayesian analysis and ABGD analysis, using seven predetermined populations (Table 2). For the comparison of eastern Pacific and western Pacific populations, the majority of genetic variation (60.54%) occurred within populations, whereas the variation among groups was 14.86% and the variation among populations within groups was 24.60%. For comparison of the three species identified in the Bayesian and maximum likelihood phylogenetic trees, the majority of genetic variation (60.48%) occurred among groups, whereas the variation among populations within groups was 2.86% and the variation within populations was 36.65% (Tables 3 and 4).

**Table 3:** Results of the two AMOVA analyses conducted using seven predetermined populations listed in Table 2; in the first analysis western Pacific populations and eastern Pacific populations were grouped separately (East vs. West) to test for genetic differentiation across the Pacific Ocean; in the second analysis the populations were grouped according to the three clades (species) recovered in the phylogenetic and ABGD analyses (three species) to test for genetic differentiation among and between the three species here recognized.

Analysis	Partitioning	d.f.	Sum of Squares	Variance component	% variation	F-statistics
East vs. West	Among groups	1	64.657	2.69095 Va	14.86	F_SC_ = 0.28894
	Among populations within groups	5	177.026	4.45348 Vb	24.6	F_ST_ = 0.39463
	Within populations	34	372.633	10.95980 Vc	60.54	F_CT_ = 0.14864
	Total	40	614.317	18.10423		
Three Species	Among groups	2	187.449	6.64190 Va	36.65	F_SC_ = 0.04517
	Among populations within groups	4	54.235	0.51845 Vb	2.86	F_ST_ = 0.31596
	Within populations	34	372.633	10.95980 Vc	60.48	F_CT_ = 0.36655
	Total	40	614.317	18.12015		

**Table 4:** Matrix of the population group comparisons results, with Φ_ST_ values (lower triangular) and associated *p* values (upper triangular).

	Washington	SouthEast	NorthEast	Canada	Alaska	SouthJapan	NorthJapan
Washington	-	0.1363	0.12976	0.37136	0.07597	0.07597	0.15598
SouthEast	0.12598	-	0.0023	0.58702	0.3918	0.21629	0.28043
NorthEast	0.18164	0.36035	-	0.45126	0.27566	0.22893	0.24482
Canada	0.11426	0	0.00293	-	-0.0752	0.68711	0.55624
Alaska	0.39258	0.00098	0.17188	0.93555	-	0.55528	0.37332
SouthJapan	0.5166	0.26562	0.14844	0.00781	0.06055	-	-0.26746
NorthJapan	0.13281	0.00586	0.02734	0.00098	0.00977	0.9541	-

### Morphological Analyses

**Fig 3:**
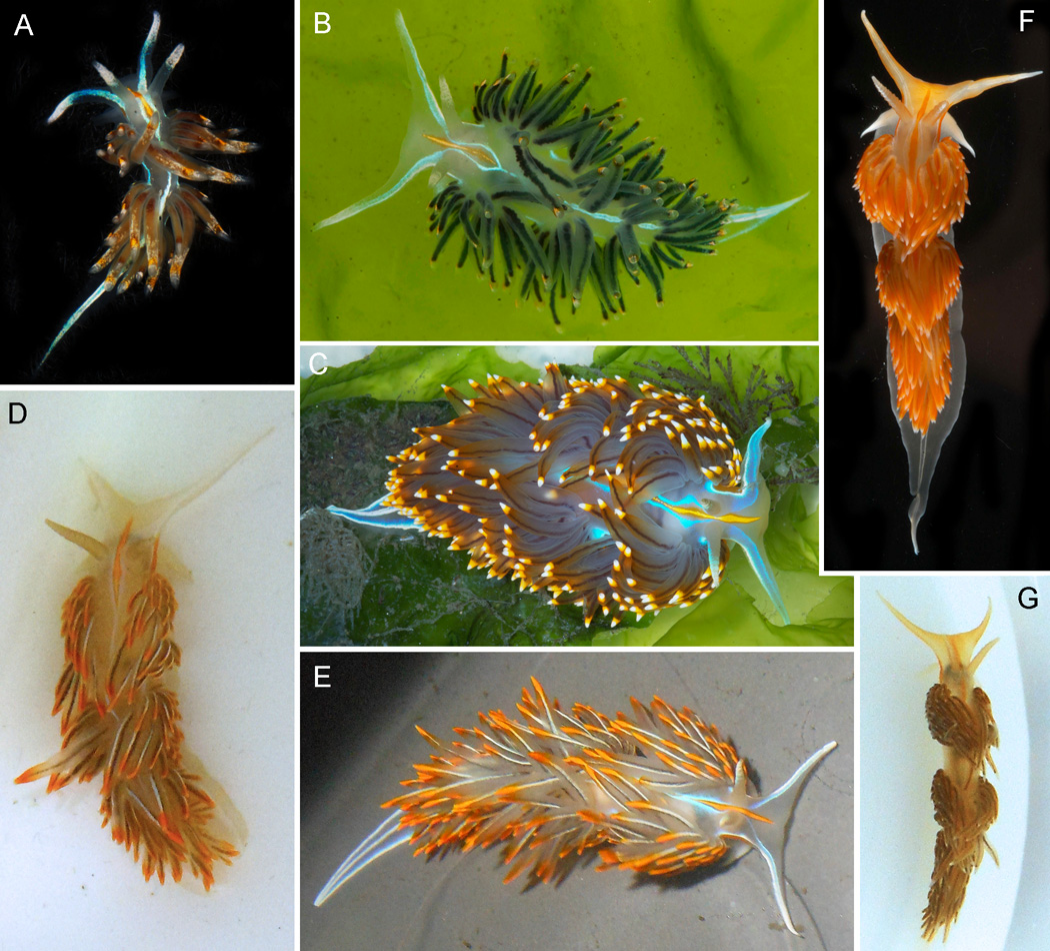
Morphological differences in specimens of *H*. *crassicornis* here examined. (A) Long Beach, California. (B) Bodega Bay, California. (C) Bodega Bay, California. (D) Sitka, Alaska. (E) Victoria, British Columbia. (F), Chiba, Japan. (G) Muroran, Japan.

External morphology was examined and compared between specimens of the three groups (species) recovered by phylogenetics, species delimitation, and haplotype network analyses (Figs 1 and 3). Consistent differences in external coloration and morphology were confirmed using images taken in the field of specimens and examining photographs from the Sea Slug Forum (www.seaslugforum.net) and www.wallawalla.edu. Both, the species found in the Sea of Japan and the species found in the Sea of Cortez through Oregon have cerata with light brown to dark brown to bright orange background color, which may or may not contain reddish to brown tipping with no apparent white stripe extending along the anterior surface of each ceras. In all three species the cerata are arranged into distinct groups, but in the species from the Sea of Japan the gaps between the groups of cerata tend to be much longer than in the other two species, making the ceratal groups much more obvious in a dorsal view; also the body of this species is much more elongate than the two eastern Pacific species. The entire body of the Sea of Japan animals exhibits an orange hue, while the specimens from the eastern Pacific show a more white or translucent body. The longitudinal strip between the rhinophores appears dark orange to an almost reddish color, while it is light orange to bright orange on specimens from the eastern Pacific. The species ranging from Alaska to northern California has light brown to dark brown to bright orange cerata with a distinct white stripe extending along the anterior surface of each ceras. These conspicuous ceratal white lines are the most characteristic external trait of this species and are never present in the species ranging from the Sea of Cortez through Oregon, making these two species easily distinguishable.

**Fig 4:**
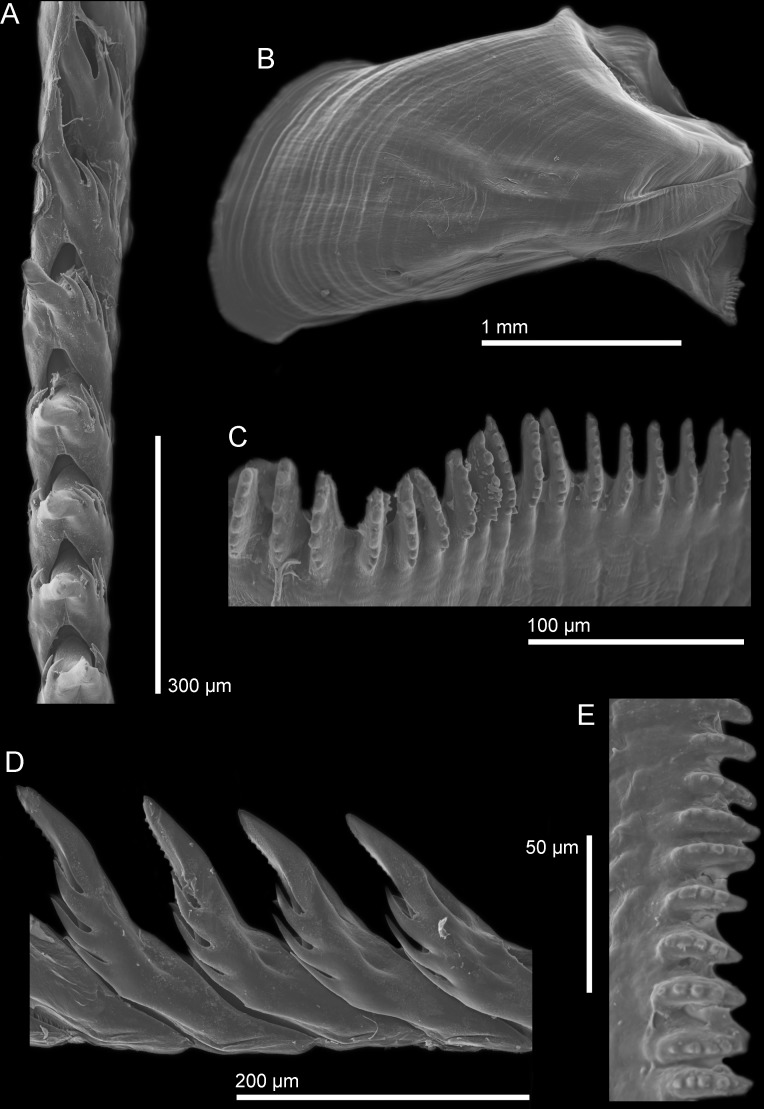
SEM images of *H*. *opalescens* from southern California. (A) Radula, dorsal view with ventral denticles of the cusp visible in some teeth. (B) Jaw (B). (C, E) Jaw masticatory border. (D) Lateral view of the radular teeth with ventral denticles of the cusp visible.

**Fig 5:**
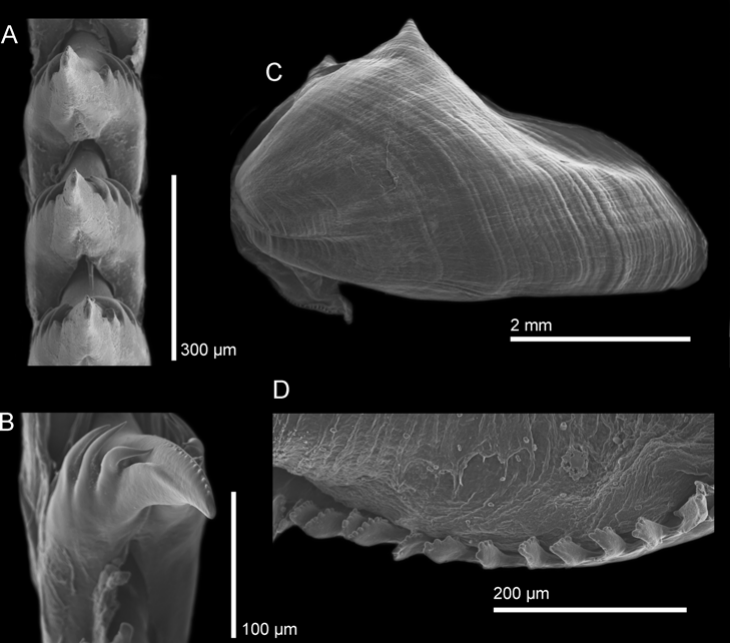
SEM images of *H*. *emurai* from Japan. (A) Radular teeth, dorsal view. (B) Radular teeth, view of the ventral side of the cusp showing the denticles. (C) Jaw. (D) Masticatory border of the jaw.

**Fig 6:**
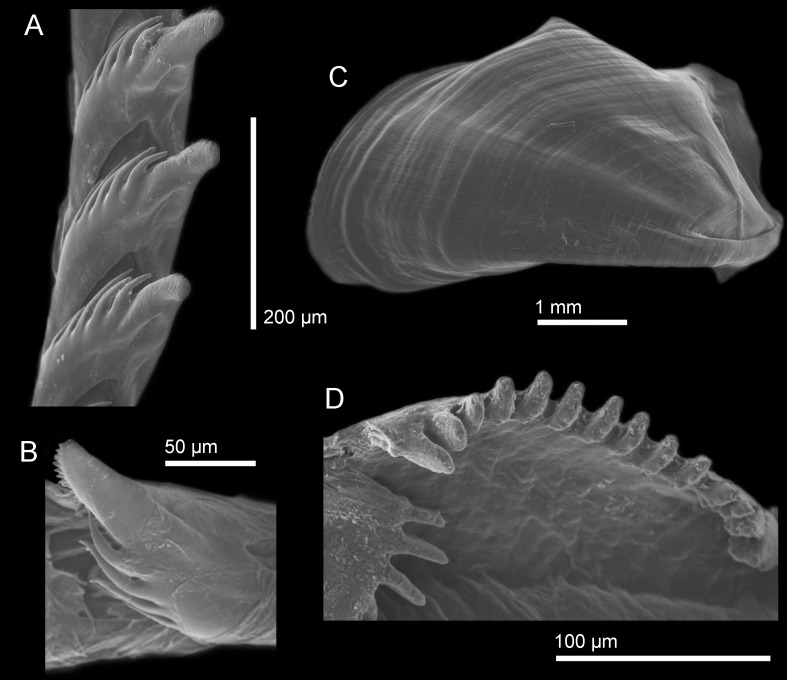
SEM images of *H*. *crassicornis* from Washington. (A) Radular teeth, dorsal view. (B) Radular tooth showing the ventral denticles of the cusp. (C) Jaw. (D) Masticatory border of the jaw.

Using SEM images, the radula formula was determined for each group (species) using at least two specimens to account for variation. The radula formula for the Sea of Japan species is 25 × (0.1.0), the radula formula for the northeastern Pacific species is 31 × (0.1.0), and the radula formula for the South Northeastern Pacific species is 28–30 × (0.1.0). The radular formula is not substantially different between the three species, however, there are very slight morphological differences in the masticatory border of each species. The South Northeastern species (Fig 4) has the largest amount of denticles, about eight, that appear as large, round projections, while the Sea of Japan species (Fig 5) has fewer denticles, about six to seven, which are not as large, and have a blunt end as opposed to a rounded end. The North Northeastern Pacific species (Fig 6) has the least amount of denticles, about four to five, which are smaller and appear more as slight bumps on the masticatory border instead of strong denticles protruding from the border.

## Discussion

Speciation is not always accompanied by morphological change, resulting in the formation of cryptic species [43], which are species physically indistinguishable from each other. Morphological divergence associated with speciation can be so subtle that differences are difficult to quantify and describe. Species that can only be distinguished *a posteriori* (after molecular data becomes available) are called pseudocryptic [44]. The existence of cryptic and pseudocryptic species constitutes a major challenge to organismal biology research and underpins the importance of modern taxonomy and systematics. The taxonomic impediment [45–48], or the lack of funding and trained taxonomists for numerous groups of organisms has pernicious consequences for conservation, and hampers progress in other scientific disciplines, such as ecology and evolutionary biology [45–48]. The advent of molecular techniques and the integrative nature of modern taxonomy have helped to solve this problem by providing more objective methodologies and faster procedures for species delineation [49]. At the same time, greater accuracy in species descriptions has revealed the existence of numerous cryptic and pseudocryptic taxa [43–44], which challenge studies that relied on pre-molecular taxonomic work. The present study is a clear example of this problem. Molecular and morphological data supports the hypothesis that the modern use of the binominal name *Hermissenda crassicornis* includes three distinct species. Therefore, experiments based on *H*. *crassicornis* as a model organism and published previous to this study might need to be re-evaluated in light of these results. Although the three species are closely related, fundamental differences in their biology might produce biases when comparing results from different studies. The results of this paper raise questions on the repeatability of past experiments based on *H*. *crassicornis*, unless the identity of the specimens can be verified, and highlight the need for careful taxonomic evaluation of model organisms collected in the wild. Because the three species in the *H*. *crassicornis* species complex are pseudocryptic and rarely overlap in range, it should be relatively straightforward to determine the identity of specimens used in previous studies, with the exception perhaps of specimens collected near the San Francisco Bay Area, where two of the species coexist.

Another implication of the results of this study is the need to conduct a thorough review of the literature to determine whether there are available names for the three species. This is done in the following paragraphs.

*Aeolis opalescens* was originally described by Cooper [50] based on specimens collected from San Diego Bay, California as “bluish white, pellucid, somewhat quadrangular, posteriorly wedge-shaped ending in a sharp point.” The foot had two anterior, “short spreading appendages and thin and flattened laterally.” The head was short with two long, acute tentacles (the lower pair replaced by the appendages of the foot), and “two erect, club-shaped rhinophores of an opaline color, with an orange stripe between them.” The “branchiae” [= cerata] were in “five pairs of fasciculi [= groups] along the upper edges of the back, each bundle of about four rows, longest above their color yellowish, with a purple or blood-red spot near the end.” There was a “rosy tint often visible from the string of ova shining through the abdominal walls.” Cooper [51] reported this species again as *Flabellina opalescens* based on additional specimens collected in San Diego as well as new records from Santa Barbara Island, differing from the original description by having olive cerata with white tips. Bergh [52] introduced the genus name *Hermissenda* for *Aeolis opalescens* Cooper, 1862 based on the original description by Cooper [50] as well as additional specimens collected by Dall in 1865 in Sitka, Alaska. Bergh [53] further expanded the description of *Hermissenda* and re-described *H*. *opalescens* providing for the first time anatomical details based on the Alaskan specimens. Cockerell [54] examined additional specimens from San Pedro, California, and reported the species from La Jolla, California, describing the external coloration as well as some anatomical features. Cockerell [54] noted some color variation between specimens found on kelp and those collected on the substrate, and indicated this species has two opal blue lines practically fused together along the dorsum, but diverging at two or more points, leaving bright orange streaks in between, as well as bright orange streaks on the sides of the head; he described the oral tentacles as opalescent blue. Cockerell & Elliot [55] studied additional specimens from San Pedro, describing the external and internal anatomy and providing drawings of the living animal. Cockerell & Elliot [55] agreed with Cockerell’s [54] assessment that his specimens from San Pedro belong to the same species as Cooper’s original animals from San Diego, but considered that the specimens from Alaska are smaller and different in coloration, without providing further details.

**Fig 7:**
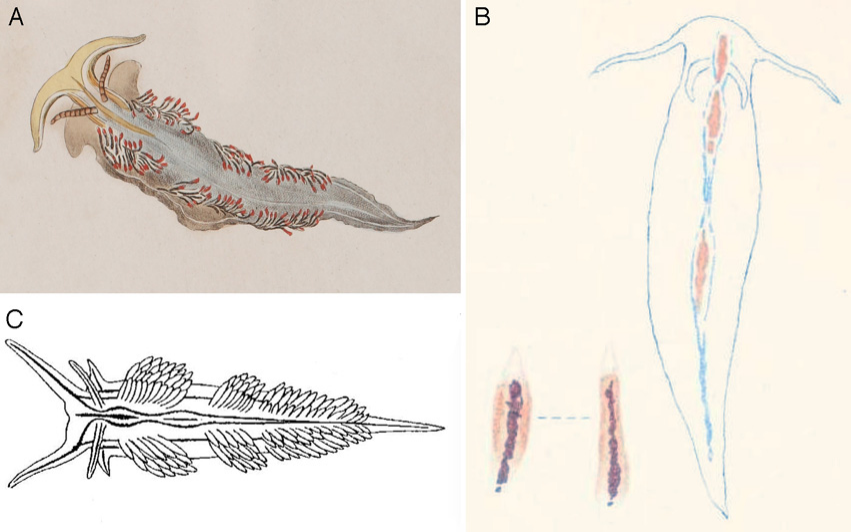
Original illustrations of the species recognized in this study. (A) *Cavolina crassicornis* by Eschscholtz [60]. (B) *Hermissenda opalescens* by Cockerell & Elliot [55]. (C) *Cuthona* (*Hervia*) *emurai* by Baba [61].

In a series of papers, O’Donoghue [56–57] and O’Donoghue & O’Donoghue [58] reported specimens of *H*. *opalescens* from the Vancouver Island region, Canada, which were described in great detail, including the internal anatomy, color variation and egg mass. O’Donoghue [56] noted his specimens had a white longitudinal line on each ceras. On a separate paper, O’Donoghue [24] rediscovered the original description of *Cavolina crassicornis* by Eschscholtz [59], and noted the similarities between his descriptions of *H*. *opalescens* and *C*. *crassicornis*. Thus, O’Donoghue [24] transferred *C*. *crassicornis* to *Hermissenda* and regarded, for the first time, *H*. *opalescens* as a junior synonym of *H*. *crassicornis*. This opinion was universally accepted [60], and the name *H*. *crassicornis* became well established in the northeastern Pacific literature [23–24]. The examination of the original description of *C*. *crassicornis* by Eschscholtz [59] (Fig 7A) from Alaska and the descriptions by O’Donoghue [56–57] of specimens from Canada reveal that their characteristics match those of the specimens here examined from Alaska to northern California, including the presence of white longitudinal lines in the cerata. Therefore, we retain the name *Hermissenda crassicornis* for this species. On the contrary, the specimens from San Diego described by Cooper [50] as *Aeolis opalescens* and subsequently illustrated by Cockerell & Elliot [55] (Fig 7B) lack white lines in the cerata and match the characteristics of the specimens here examined from the Sea of Cortez to northern California. Thus, we re-introduced the name *Hermissenda opalescens* for this second species. Baba [61] described *Cuthona* (*Hervia*) *emurai* based on specimens collected in Niigata, Niigata Prefecture (Sea of Japan). The species was described as follows: “The ground-colour of the body is of a pale (fleshy) yellow. Along the mid-dorsal region there run two bluish bilateral lines which pass forward and run right up the rhinophores and oral tentacles; posteriorly they converge to the tip of the tail. A broken mid-dorsal vermilion line runs about half way down from the head. The sides of the body are each marked with two lines running parallel with each other, the upper bluish and the lower shorter and vermilion. The branchial papillae [= cerata] are chocolate-coloured with usually a white vein and a vermilion marking immediately below the whitish tip, sometimes a white broken vein running up to the tip. The antero-lateral tentaculiform processes of the foot are each marked with a bluish line.” Years later, McDonald [25] proposed that *Cuthona emurai* was a color variation of the *Hermissenda crassicornis* (under *Phidiana*) and formally synonymized these two species. Based on Baba’s [61] original description and illustrations of the radula, the jaws and the external morphology (Fig 7C), which closely match those of the specimens from the Sea of Japan here examined, as well as the fact that *Cuthona emurai* was described from Japan, we propose using the name *Hermissenda emurai* for the species here recognized from in the Sea of Japan.

Miller [62] and McDonald [25] placed *Hermissenda crassicornis* in the genus *Phidiana*. However, this opinion was not accepted by other authors in subsequent publications. A recent phylogenetic analysis of aeolid nudibranchs [63] as well as the present study show species of *Phidiana* and *Hermissenda* in different clades. Thus, we maintain *Hermissenda* as distinct from *Phidiana*.

## Conclusions

The model organism *Hermissenda crassicornis* is a complex of three pseudocryptic species. Because the name *H*. *crassicornis* was introduced for specimens collected in Alaska, this name is retained for the northeast Pacific species, which occurs in Alaska, the Pacific coast of Canada, Washington, Oregon as well as Point Reyes, Northern California (based on the material here examined). The name *H*. *opalescens*, originally introduced from Southern California, is reinstated for the southwestern species, found from the Sea of Cortez, Mexico to Bodega Bay, Northern California. Finally, the name *H*. *emurai*, introduced for Japanese specimens, is maintained for the northwestern species, found in Japan and in the Russian Far East. Close morphological examination of the three species revealed consistent morphological differences that can be used for identification in the field. This is particularly important where *H*. *crassicornis* and *H*. *opalescens* overlap in range, between Point Reyes and Bodega Bay. All specimens of *H*. *crassicornis* examined have white, longitudinal lines on their cerata, which are absent in *H*. *opalescens*. On the other hand, *H*. *emurai* is also distinguishable by having the cerata arranged in distinct groups and a more orange overall coloration.
